# The cohesin modifier ESCO2 is stable during DNA replication

**DOI:** 10.1007/s10577-023-09711-1

**Published:** 2023-01-28

**Authors:** Allison M. Jevitt, Brooke D. Rankin, Jingrong Chen, Susannah Rankin

**Affiliations:** 1 Cell Cycle and Cancer Biology Program, Oklahoma Medical Research Foundation, Oklahoma City, OK 73104 USA; 2 Department of Cell Biology, University of Oklahoma Health Sciences Center, Oklahoma City, OK 73104 USA

**Keywords:** Chromosome cohesion, DNA replication, ESCO2, E3 ubiquitin ligase, *Xenopus laevis* egg extract, Cell cycle

## Abstract

Cohesion between sister chromatids by the cohesin protein complex ensures accurate chromosome segregation and enables recombinational DNA repair. Sister chromatid cohesion is promoted by acetylation of the SMC3 subunit of cohesin by the ESCO2 acetyltransferase, inhibiting cohesin release from chromatin. The interaction of ESCO2 with the DNA replication machinery, in part through PCNA-interacting protein (PIP) motifs in ESCO2, is required for full cohesion establishment. Recent reports have suggested that Cul4-dependent degradation regulates the level of ESCO2 protein following replication. To follow up on these observations, we have characterized ESCO2 stability in *Xenopus* egg extracts, a cell-free system that recapitulates cohesion establishment in vitro. We found that ESCO2 was stable during DNA replication in this system. Indeed, further challenging the system by inducing DNA damage signaling or increasing the number of nuclei undergoing DNA replication had no significant impact on the stability of ESCO2. In transgenic somatic cell lines, we also did not see evidence of GFP-ESCO2 degradation during S phase of the cell cycle using both flow cytometry and live-cell imaging. We conclude that ESCO2 is stable during DNA replication in both embryonic and somatic cells.

**Supplementary Information:**

The online version contains supplementary material available at 10.1007/s10577-023-09711-1.

## Introduction

The tethering together of sister chromatids during DNA replication depends in part on acetylation of the SMC3 subunit of cohesin, which renders the complex resistant to removal from chromatin by the WAPL protein (Unal et al. 2008; Zhang et al. 2008; Sutani et al. 2009). In vertebrates, SMC3 acetylation is achieved by one of two related acetyltransferase enzymes, ESCO1 and ESCO2 (Hou and Zou 2005). Using the *Xenopus* egg extract system, we previously showed that ESCO1 is developmentally regulated and not present at functional levels until after zygotic transcription begins (Lafont, Song, and Rankin 2010). In egg extracts, therefore, ESCO2 is the sole cohesin acetyltransferase required for cohesion between sister chromatids, and depletion of ESCO2 from egg extract results in significant loss of cohesion (Song et al. 2012; Lafont, Song, and Rankin 2010).

Multiple reports suggest cell cycle-dependent fluctuations in ESCO2 protein levels, although there are conflicting reports about the precise timing. Some reports indicate that ESCO2 levels peak during S phase (Minamino et al. 2018) and are thus low prior to mitotic entry, while others have suggested that ESCO2 is degraded during M phase (Lelij et al. 2009; Hou and Zou 2005). ESCO2 has also been reported to be stabilized by interaction with the MCM helicase during replication licensing, suggesting a third, perhaps indirect, level of stability control (Minamino et al. 2018; Bender et al. 2019; Ivanov et al. 2018).

ESCO2 protein levels are controlled at least in part by ubiquitin-dependent proteolysis (Lafont, Song, and Rankin 2010). The anaphase-promoting complex (APC) is an E3 ubiquitin ligase that has numerous substrates, including some that are degraded at mitotic exit, and others that continue to be recognized through G1 (Davey and Morgan 2016). As in other APC targets, a degron sequence in ESCO2 mediates recognition and modification by the APC when it is bound to the G1 specificity factor called Cdh1 (Visintin, Prinz, and Amon 1997; Davey and Morgan 2016; Lafont, Song, and Rankin 2010). Mutation of this sequence stabilizes ESCO2, preventing its degradation in the presence of active APC^Cdh1^ (Lafont, Song, and Rankin 2010).

It has been suggested that degradation of ESCO2 is also controlled by a second E3 ubiquitin ligase, the CUL4-DDB1 complex via the specificity factor DCAF1 (DDB1 and CUL4 associated factor 1, also called VprBP), resulting in post-replicative degradation prior to M phase (Minamino et al. 2018). Together, these reports suggest an interesting dual regulation of ESCO2 by proteolysis: in G1 by the APC, and during S phase by the CUL4-DDB1-DCAF1^VprBP^ complex.

To better understand the regulation of ESCO2 protein turnover, we set out to identify the degron that might mediate recognition of ESCO2 by CUL4-DDB1-DCAF1^VprBP^. To this end, we analyzed ESCO2 stability, utilizing the *Xenopus* egg extract system, which is a powerful tool to investigate CUL4-dependent mechanisms (Jin et al. 2006; Arias and Walter 2005; Arias and Walter 2006; Havens et al. 2012; Havens and Walter 2009). Our results indicate that ESCO2 is stable during DNA replication in the egg extract system. We also tested ESCO2 stability in cultured somatic cells, where we saw no evidence of degradation after G1 phase of the cell cycle. Our data suggest that accumulation of ESCO2 in the absence of CUL4-DDB1-DCAF1^VprBP^ seen previously may occur through indirect mechanisms.

## Results

Extracts prepared from the eggs of the frog *Xenopus laevis* are stockpiled with sufficient proteins for the replication of thousands of nuclei per microliter, making this system ideal for the study of DNA replication-dependent events in vitro (Jevitt and Rankin 2022; Rankin 2019). Demembranated sperm heads added to the extract are assembled into nuclei through the recruitment of membrane vesicles from the extract and the import of nuclear and chromatin proteins. Regulated DNA replication and replication-dependent events such as cohesion establishment and CUL4-dependent degradation all occur in these in vitro assembled nuclei (Arias and Walter 2005; Arias and Walter 2006; Havens and Walter 2009; Shintomi and Hirano 2017; Losada, Hirano, and Hirano 1998; Song et al. 2012).

**Fig. 1:**
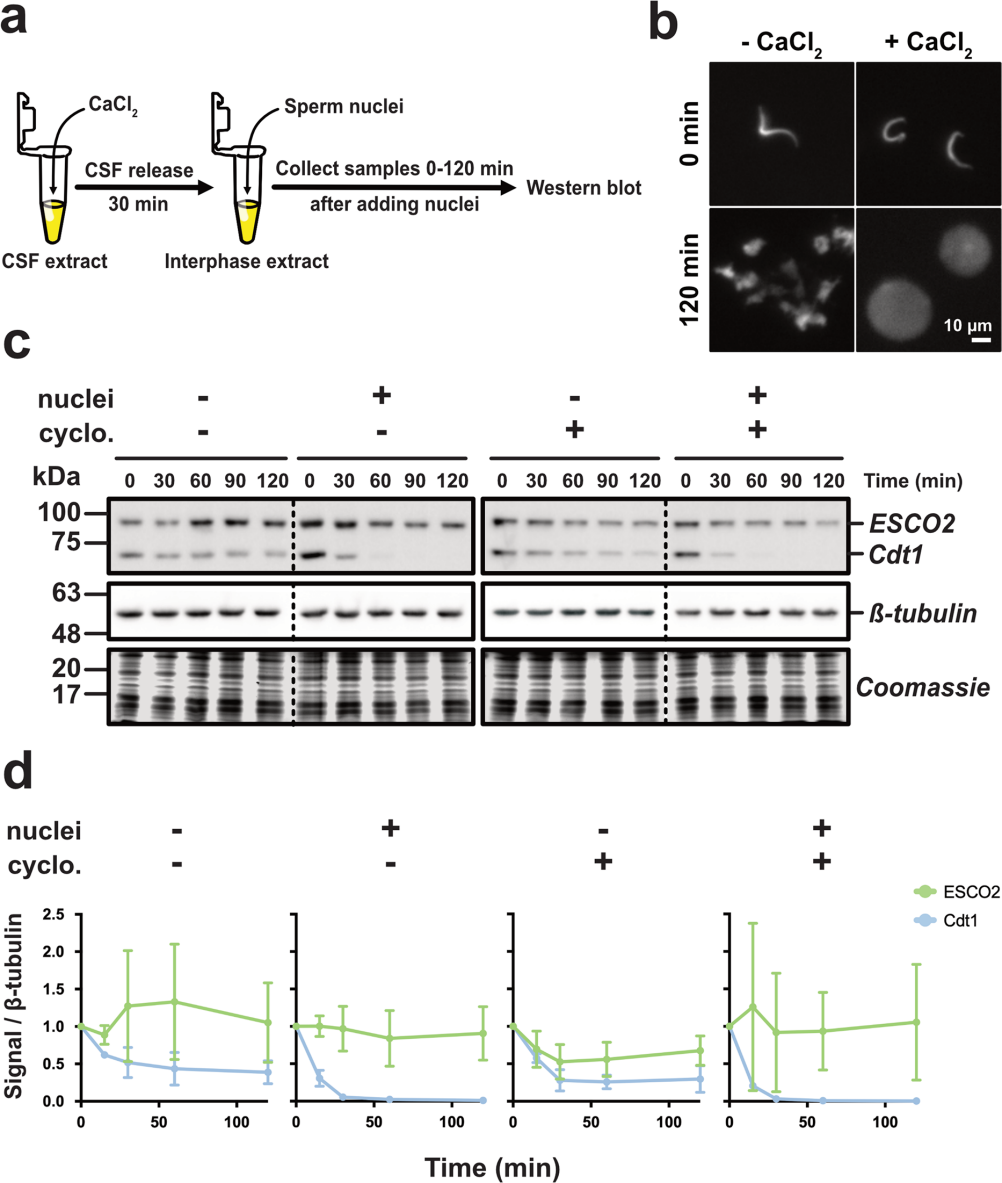
ESCO2 protein level remains constant during DNA replication. **a** Approach. CSF-arrested extract was induced to enter interphase by the addition of calcium (CaCl_2_). Thirty minutes later, sperm nuclei (2300/μl, final) were added and aliquots were collected at the indicated times. **b** Representative fluorescence images of nuclei sampled right after addition into extracts (0 min) and after a 120 min incubation. Nuclear morphology associated with mitotic exit at bottom right was observed after addition of calcium. DAPI stain marks nuclei. **c** Immunoblot analysis. Reaction samples were analyzed by immunoblot for the indicated proteins. Cyclo: Cyclohexamide was added where indicated to prevent protein translation. Solid outlines denote membrane fragments that were processed separately. Dotted lines denote where gel images were cropped. ESCO2 and Cdt1 were analyzed on the same membrane fragment. *β*-tubulin served as a loading control. The foot of the gel was collected and stained with Coomassie as an additional loading control. **d** Quantification of results. The representative experiment shown in **c** was repeated three times and the results were plotted as a fraction of the remaining signal for the indicated proteins normalized to the *β*-tubulin signal for each sample. Error bars = SD

Here we set out to explore the relationship between ESCO2 protein turnover and DNA replication in detail using the *Xenopus* egg extract system, which can easily be manipulated and is intrinsically synchronized. A previous report using egg extract suggests that ESCO2 dissociates from chromatin during S phase progression, but did not directly address the possibility of degradation (Higashi et al. 2012). Here we test directly for degradation. To do this, we started with cytostatic factor (CSF) arrested extract, a membrane-containing preparation that is suited for the study of DNA replication and replication-dependent events that require nuclear assembly and import (Gillespie, Gambus, and Blow 2012). The extract was first induced to enter interphase by the addition of calcium, which mimics the fertilization reaction (Fig. 1a), then supplemented either with nuclei (2300/μl) or sham vehicle (buffer). Successful mitotic exit and subsequent nuclear assembly were confirmed by analyzing nuclear morphology, assessing for both nuclear envelope formation and chromosome decondensation (Fig. 1b) (Gillespie, Gambus, and Blow 2012). Samples were collected from the reactions and the level of endogenous ESCO2 in the extract was assessed over time. We found that ESCO2 levels were not impacted by the presence of nuclei in the extract (Fig. 1c–d). In contrast, the replication licensing factor Cdt1, a previously characterized CUL4 substrate, showed clear nuclei-dependent degradation in the same extract. In control samples without nuclei, Cdt1 levels decreased only slightly over the 2-h experiment, as previously reported, while in the presence of nuclei, Cdt1 was largely depleted by 60 min (Arias and Walter 2005). While we occasionally saw slight reduction in ESCO2 level, this was unaffected by the presence of nuclei, and thus unrelated to DNA replication. We saw only modest dissociation of ESCO2 from chromatin during DNA replication (Supplementary Fig. 1), as previously (Song et al. 2012; Lafont, Song, and Rankin 2010).

Although there is virtually no transcriptional activity in egg extract (Newport and Kirschner 1982), the extract likely contains endogenous maternal mRNA stores (Murray and Kirschner 1989). To rule out the possibility that the new translation of ESCO2 from maternal mRNA might mask our ability to detect protein loss, we blocked translation by adding cycloheximide to the extract. This treatment did not impact ESCO2 levels during S phase (Fig. 1c–d). We conclude from this experiment that ESCO2 is not destabilized by DNA replication in egg extract, though the CUL4-dependent degradation machinery is active.

**Fig. 2:**
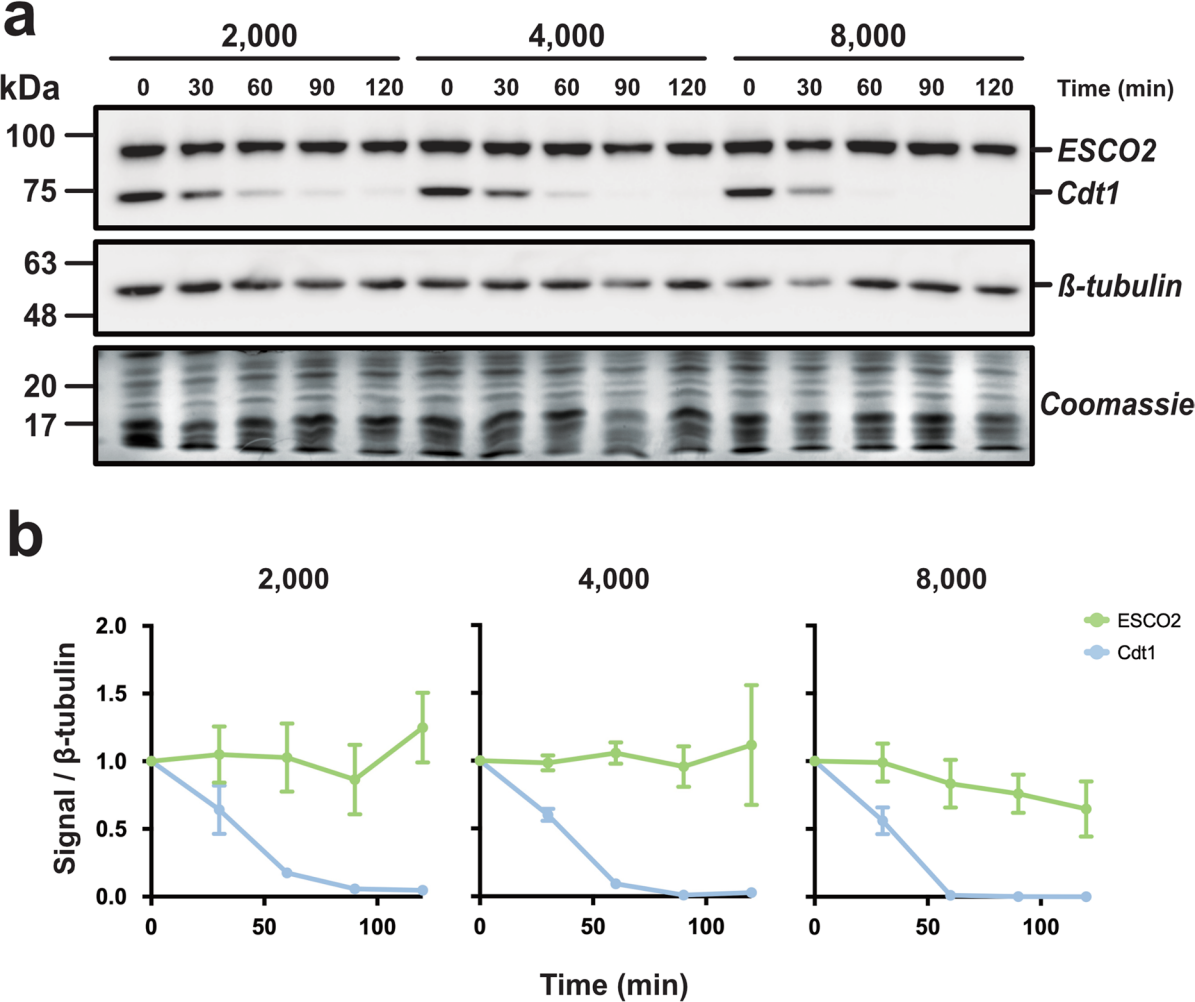
ESCO2 stability is unaffected by changes in the nucleus:cytoplasm ratio. **a** Immunoblot analysis. Interphase extract was supplemented with the indicated concentrations of sperm nuclei (2000, 4000, or 8000 sperm nuclei/μl). Samples were collected at the indicated time points after the addition of sperm nuclei and were processed for immunoblot as in Fig. 1c. Solid outlines denote membrane fragments that were processed separately. **b** Quantification of results. The representative experiment shown in **a** was repeated three times and the results were plotted as a fraction of the remaining signal for the indicated proteins normalized to the *β*-tubulin signal for each sample. Error bars = SD

Although we saw no clear decrease in ESCO2 level during DNA replication in the egg extracts, we wondered whether ESCO2 stability might be sensitive to the density of DNA replication, or the number of nuclei in the extract, perhaps being more efficiently degraded at increased nuclear density. To test this, we performed a titration experiment in which extract was supplemented with increasing concentrations of nuclei and analyzed the level of endogenous ESCO2 protein over time (Fig. 2). We found that increasing the nuclei concentration had no significant impact on ESCO2 protein levels. In fact, increasing the concentration of nuclei well above the highest nucleus:cytoplasm (N/C) ratio found in embryos during induction of zygotic transcription, estimated to be ~4000 nuclei/μl (Newport and Kirschner 1982), had no impact on ESCO2 stability. Even at 8000 nuclei/μl, the ESCO2 levels were stable over the course of the experiment. In contrast, the degradation of Cdt1 was easily detectable at all nuclear densities, and enhanced by the presence of additional nuclei in the extract (Fig. 2b). In the presence of 2000 nuclei/μl, degradation was essentially complete by 90 min, while in the presence of 8000 nuclei/μl, Cdt1 was undetectable at 60 min. We conclude from this experiment that ESCO2 degradation cannot be stimulated by increasing the N/C ratio, although Cdt1 degradation was enhanced under these conditions.

**Fig. 3:**
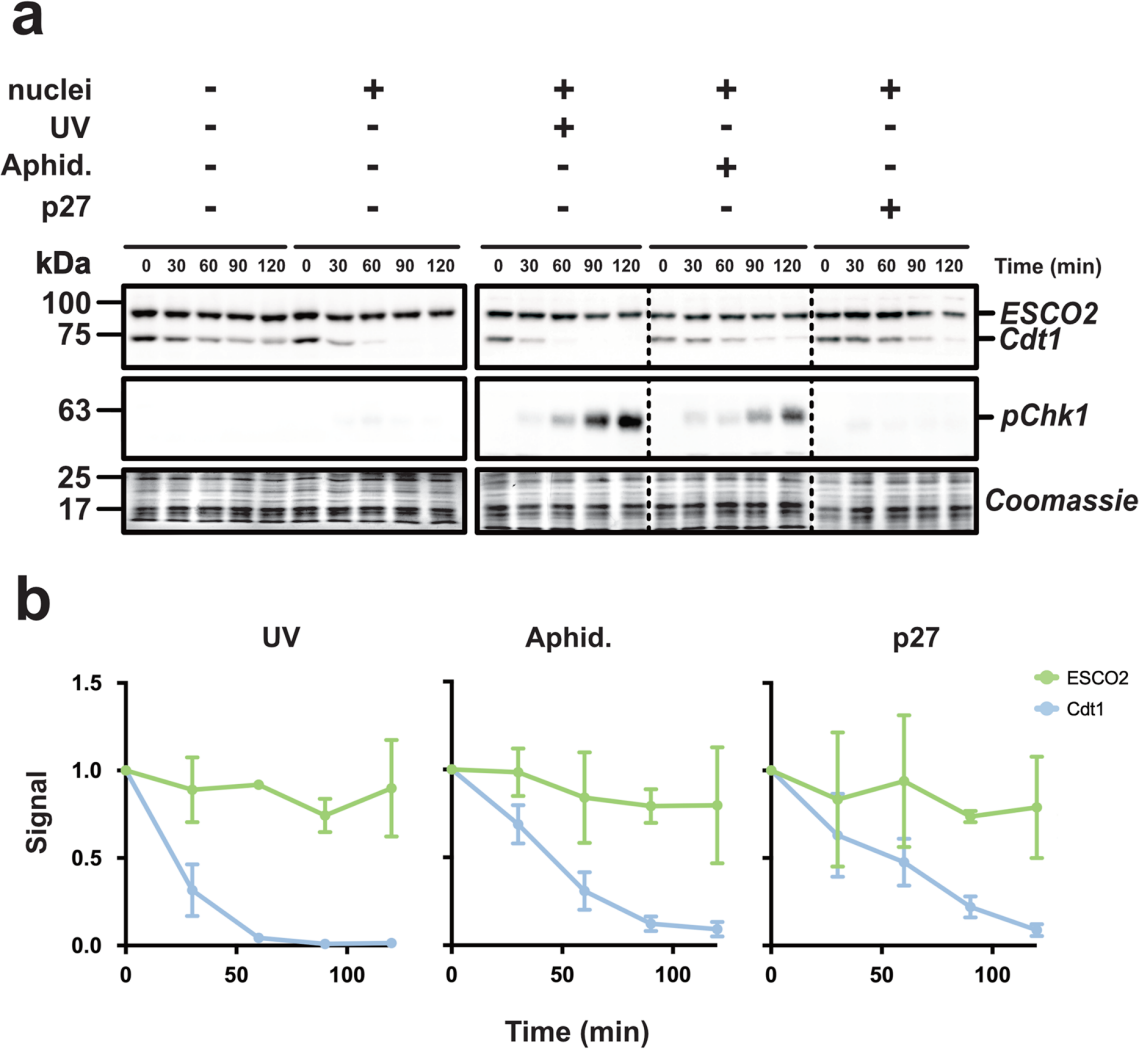
ESCO2 is stable during DNA damage signaling. **a** Immunoblot analysis. Reactions were assembled as in Fig. 1 with the indicated modifications. UV: sperm were UV treated before they were added to the extract. Aphid: the DNA replication inhibitor aphidicolin was added before the addition of nuclei. p27: Recombinant p27 protein was added to the extract before the addition of nuclei. Samples were collected at the indicated times and probed by immunoblot for the indicated proteins. Antibody specific for phosphorylated Chk1 kinase (pChk1) was used to confirm DNA damage signaling. Solid outlines denote membrane fragments that were processed separately. Dotted lines denote where blot images were cropped. **b** The representative experiment shown in **a** was repeated three times and the results were plotted as a fraction of remaining signal for the indicated proteins for each sample. Error bars = SD

In budding yeast, the stability of the ESCO2 ortholog Eco1p is modulated by DNA damage signaling (Lyons and Morgan 2011). To determine whether vertebrate ESCO2 stability might be regulated in response to DNA damage signaling, we tested two conditions known to activate the DNA damage response in egg extracts (Fig. 3). First, we added the DNA polymerase inhibitor aphidicolin to the reaction. This drug causes the uncoupling of polymerase *α* from the replicative helicase, resulting in the formation of single-stranded DNA, which in turn activates the DNA damage response (Byun et al. 2005; Hekmat-Nejad et al. 2000; Recolin, Laan, and Maiorano 2012). In addition to the aphidicolin treatment, we also tested the effect of a DNA damage response generated by the addition of UV-irradiated sperm to the reaction (Kumagai, Yakowec, and Dunphy 1998). In both conditions, DNA damage signaling was verified by monitoring the phosphorylation of Chk1 checkpoint kinase (Walworth and Bernards 1996) (Fig. 3). Under both of these conditions, ESCO2 levels remained stable. As seen previously, Cdt1 degradation was largely unaffected by UV damage of the sperm nuclei, and was inhibited by the presence of aphidicolin. The effect of aphidicolin is consistent with the requirement for ongoing DNA replication for Cdt1 degradation shown previously (Havens and Walter 2009). Similarly, inhibition of DNA replication by the addition of the Cdk inhibitor p27 also slowed Cdt1 degradation (Fig. 3). We conclude from this experiment that ESCO2 stability is largely unaffected by DNA damage signaling, whether or not DNA replication is active, consistent with the model that ESCO2 and Cdt1 stability are controlled by different mechanisms. We conclude from this experiment that ESCO2 stability is largely unaffected by DNA damage signaling, whether or not DNA replication is active, consistent with the model that ESCO2 and Cdt1 stability are controlled by different mechanisms.

Reliance on different specificity factors may explain the different sensitivity of ESCO2 and Cdt1 to CUL4-dependent degradation in egg extract. The CUL4 ubiquitin ligase can be activated by one of a number of DCAF subunits, which confer substrate specificity to the complex (Jin et al. 2006). Cdt1 is recognized by CUL4 when it is activated by DCAF2^Cdt2/Dtl^, and Cdt1 degradation requires interaction with chromatin-bound PCNA through a specific motif called a PIP-degron, explaining the requirement for DNA replication (Havens and Walter 2009). Unlike Cdt1, ESCO2 has been proposed to be ubiquitinated by CUL4 activated by DCAF1^VprBP^, which is not well characterized in the *Xenopus* egg extract system and likely acts independently of PCNA (Minamino et al. 2018). It is possible that DCAF1^VprBP^-dependent degradation is not fully active until later in development, or has developmentally regulated changes in specificity. Although the replication regulator MCM10 has been reported to be degraded through CUL4-DCAF1^VprBP^ in response to UV damage (Kaur et al. 2012), we found endogenous MCM10 to be stable in egg extracts, even in the presence of active DNA damage signaling (Supplementary Fig. 2). Because proteomic analyses suggest that the DCAF1^VprBP^ protein level is relatively constant during early *Xenopus* development, it is unlikely that CUL4 activity is controlled by changes in DCAF1^VprBP^ expression at this time (Peshkin et al. 2019) (Supplementary Fig. 3). Fully understanding the role of DCAF1^VprBP^ during early embryogenesis will require additional detailed studies beyond the scope of this current investigation.

Because we were unable to detect ESCO2 degradation during or following DNA replication, and because we could not with certainty identify an appropriate control for DCAF1^VprBP^-dependent degradation in the *Xenopus* embryonic system, we further investigated ESCO2 stability in somatic cells. To do this, we created stable HeLa cell lines in which a GFP-ESCO2 transgene is under the control of a tetracycline-inducible promoter. The advantage of this approach is that GFP-ESCO2 levels can be monitored in live, asynchronously growing cells, while previous experiments were performed with thymidine- or nocodozole-based synchronization protocols. Synchronization protocols using drug treatments can have unwanted cell cycle impacts such as replication stress from stalled replication forks, an imbalance of cell cycle regulatory proteins, or DNA damage (Ligasová and Koberna 2021). In addition, synchronization with thymidine results in artificial accumulation of APC^Cdh1^ targets such as ESCO2, making assessment of instability difficult.

**Fig. 4:**
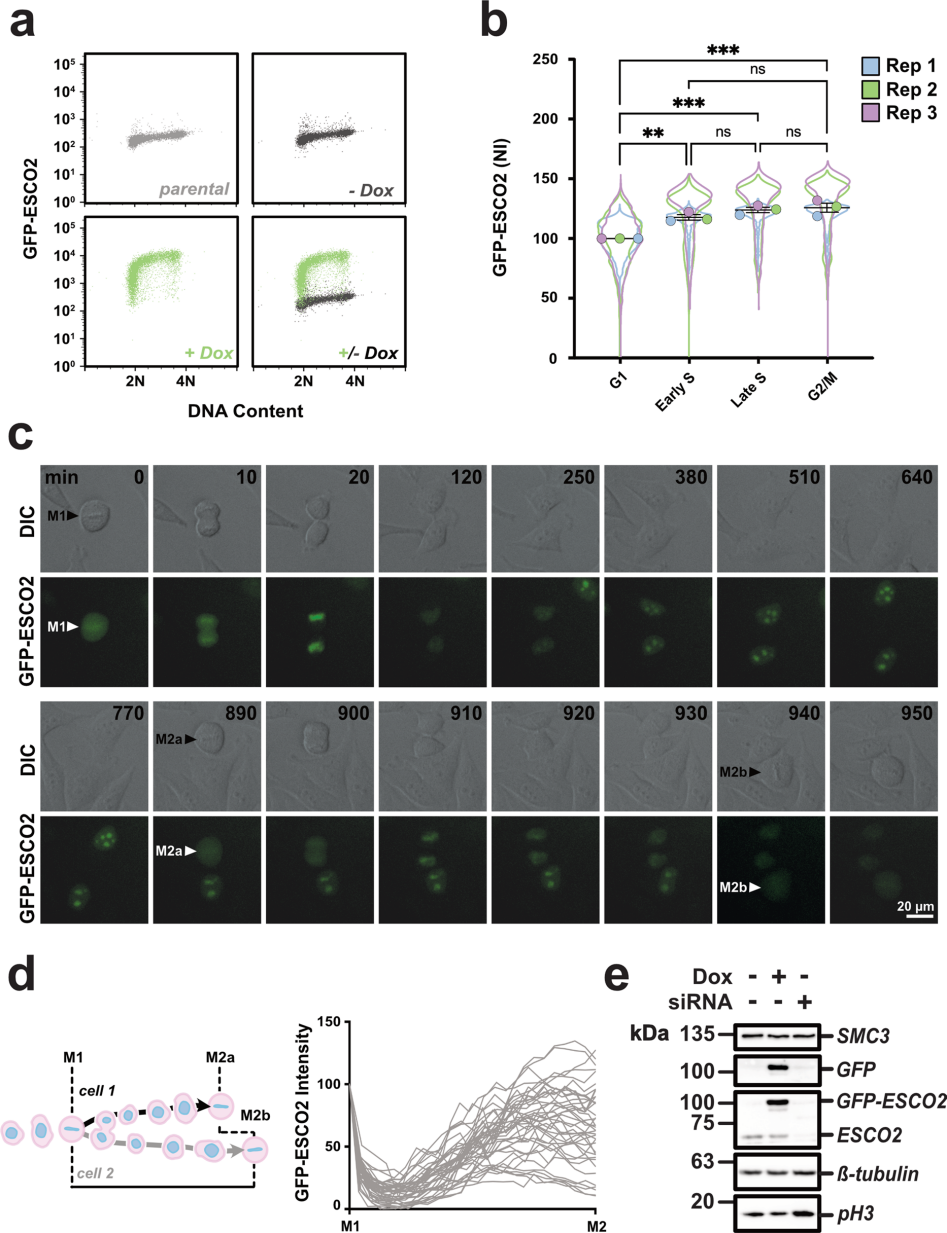
ESCO2 levels in cultured somatic cells. **a** Flow cytometry. Live non-extracted HeLa cells expressing GFP-ESCO2 were collected and analyzed for GFP levels and DNA content. Parental and uninduced GFP-ESCO2 (no doxycycine added) cell lines are also shown. *n* ≧ 5500. **b** Quantification of GFP-ESCO2 levels during cell cycle progression. The flow cytometry experiment shown in **a** was repeated three times and are shown together as a SuperPlot (Lord et al. 2020). Mean values from each replicate, normalized to their G1 mean value, are plotted together. NI, normalized intensity. Error bars = SEM. ns, not significant; **, *p* < 0.01; ***, *p* < 0.001; ****, *p* < 0.0001. **c** Time-lapse imaging. Shown are selected frames from time-lapse imaging of HeLa cells expressing GFP-ESCO2. Intervals were chosen to highlight details of cell division. Time elapsed (in minutes) since metaphase is indicated by numbers in black font. The complete movie is shown in Supplementary Video 1. **d** Quantification of GFP signal. Schematic at left shows how GFP fluorescence intensity was scored across a complete cell division cycle, from an initial metaphase (M1) to metaphase 2 (M2) for each daughter cell, and graphed in aggregate at right. *n* = 20 M1 cells, 40 M2 cells analyzed. **e** Immunoblot analysis. The cell line used in **a**–**d** was treated either with doxycycline to induce transgene expression or siRNA to deplete the endogenous protein, and cell lysates were analyzed by immunoblot for the indicated proteins. Solid outlines denote membrane fragments that were processed separately. SMC3, a subunit of cohesin, and *β*-tubulin served as loading controls. A replicate blot was prepared and probed for GFP. Phosphorylated H3 (pH3), a marker of mitotic cells, confirmed increased mitotic index in cells depleted of ESCO2 seen previously (Alomer et al. 2017)

Asynchronous cultures of cells expressing GFP-ESCO2 were collected and analyzed by flow cytometry to assess ESCO2 levels relative to DNA content, a marker of S phase progression (Fig. 4a–b). In this cell population, we found that ESCO2 levels were constant over the course of DNA replication, with no statistically significant differences between cells in early or late S phase. Levels were significantly lower in G1 compared to G2/M (Fig. 4b) consistent with previous work showing that ESCO2 is targeted for degradation by APC^Cdh1^ (Lafont, Song, and Rankin 2010). We conclude from this experiment that ESCO2 is stable during S phase and that APC-dependent modification in G1 likely accounts for all readily detectable ESCO2 degradation during cell cycle progression.

To further characterize ESCO2 stability during cell cycle progression, we analyzed our GFP-ESCO2 cell line by time-lapse microscopy (Fig. 4c, Supplementary Video 1, Supplementary Video 2). As expected, the GFP signal was nuclear in interphase and dispersed from chromatin as cells entered M phase (Hou and Zou 2005). Consistent with our previous results, GFP-ESCO2 also accumulated in nucleoli (Bender et al. 2019). This may be due in part to overexpression, or to the presence of the GFP tag which is known to partition to this location (Martin et al. 2015). To quantify ESCO2 turnover, we measured the total cellular GFP signal as single cells progressed from metaphase to the subsequent metaphase of each resulting daughter cell (Fig. 4d). Consistent with the flow cytometry data, the GFP-ESCO2 signal was high in metaphase, and dropped after the cell division, with minimal levels attained approximately 160 min after initiation of anaphase. As cells further progressed through the cell cycle, the GFP-ESCO2 signal rose and remained elevated until the next metaphase. We saw no clear evidence of ESCO2 loss after the initial drop at anaphase (Fig. 4d). Thus by both by flow cytometry and image analysis of individual cells, we saw no evidence of a decrease in ESCO2 accompanying DNA replication. We cannot rule out the possibility that overexpression affected turnover, but note that the anticipated APC-dependent loss of ESCO2 in G1 was readily detected. Immunoblot analysis indicated that in our cell line, GFP-ESCO2 was expressed at ~6.5 times the level of endogenous ESCO2 (Fig. 4e), with some variability between cells (Supplementary Video S2). We conclude from these experiments that ESCO2 is largely stable during S phase in somatic cells.

## Discussion

We have tested ESCO2 stability during DNA replication, both by assessing the endogenous protein in *Xenopus* egg extract and by using a stably expressed fusion protein in cultured somatic cells. Although it has been proposed that ESCO2 is degraded by CUL4-dependent mechanisms during or shortly after S phase, here we find that ESCO2 is stable during DNA replication in both systems.

There are several possible explanations for the different conclusions drawn here and those made previously. Previous work showed that ESCO2 levels were elevated (compared to controls) in mitotic cells following depletion of DCAF1^VprBP^, but ESCO2 was not shown to be directly modified by the CUL4 ubiquitin ligase (Minamino et al. 2018). The increase in ESCO2 in the absence of DCAF1^VprBP^ may be an indirect result of the impact of DCAF1^VprBP^ on cell cycle progression. DCAF1^VprBP^ was originally characterized based on its interaction with lentiviral R proteins, which is thought to alter cell cycle progression, although the underlying mechanism is not known. DCAF1^VprBP^ does have cell cycle impacts independent of viral infection (Han et al. 2020). In unperturbed cells, the tumor suppressor NF2^Merlin^, an ezrin-moesin-radixin (ERM) family protein that is mutated in neurofibromatosis type 2, binds to and inhibits the nuclear CUL4-DCAF1^VprBP^ complex (Trofatter et al. 1993; Rouleau et al. 1993; Cooper et al. 2011). Depletion of CUL4-DCAF1^VprBP^ phenocopies the loss of Merlin function, suggesting that CUL4-DCAF1^VprBP^ normally has an anti-proliferative function. In other models, such as T cell development, DCAF1^VprBP^ is required for cell cycle entry (Guo et al. 2016). The ubiquitination targets of this complex that modulate proliferative activity have not been identified, although certain transcription factors may be important (Wang et al. 2017). In addition to its role in protein ubiquitination, DCAF1^VprBP^ also has non-proteolytic impacts on several proteins, including the p53 tumor suppressor, the FoxM1 transcription factor, and the SAMHD1 viral restriction factor (Nakagawa, Mondal, and Swanson 2013). The myriad activities of the CUL4-DDB1-DCAF1^VprBP^ complex make understanding its precise contribution to ESCO2 stability unclear. Fully assessing the function and activity of CUL4-DDB1-DCAF1^VprBP^ in embryonic extracts is an important topic beyond the scope of this current work.

We initially set out in this work to identify degrons in the ESCO2 protein that could promote protein turnover during or shortly after S phase, perhaps controlling cohesin function in G2 cells. We have found the ESCO2 protein to be unaffected by DNA replication in egg extract, and in somatic cells only obviously reduced in G1, most likely through the previously reported APC degron in the ESCO2 N terminus (Lafont, Song, and Rankin 2010). It remains possible that S phase-related degradation of ESCO2 is developmentally controlled or cell-type specific, perhaps occurring through mechanisms that are not yet established in the early frog embryo or elaborated later in development. However, we found no evidence for significant loss of ESCO2 during DNA replication in unperturbed somatic cells or in embryonic extract. Although previous work makes clear that ESCO2 makes complex interactions with the DNA replication machinery (Song et al. 2012; Bender et al. 2019; Ivanov et al. 2018; Higashi et al. 2012), whether ESCO2 has functions after DNA replication remains unclear at this time. Our current best model is that ESCO2 is unloaded from chromatin after MCM2-7 helicase release, and then targeted for degradation by the APC in G1, only accumulating to functional levels again when APC^Cdh1^ is inactivated in S phase.

It is difficult to reconcile our observations with previous suggestions that ESCO2 is protected from degradation by interaction with the MCM complex. If such a model were true, then soluble ESCO2 should be intrinsically unstable, which we did not see. In a more nuanced model, only ESCO2 that has been previously bound and unloaded from chromatin would be susceptible to degradation. We saw no evidence for this in somatic cells or egg extract. In both egg extract and somatic cells, MCM unloading from chromatin appears to precede ESCO2 dissociation, suggesting that ESCO2 association with chromatin is sustained through interaction with different partners later in replication (Ivanov et al. 2018; Higashi et al. 2012). Interestingly, ESCO2 mutants that cannot interact with the MCM complex do seem to have reduced stability, but this may be unrelated to CUL4 (Minamino et al. 2018; Bender et al. 2019). Fluorescence recovery experiments in somatic cells indicate that ESCO2 is retained in the nucleus, but has shorter residence time on chromatin in G2 after DNA replication is complete (Ivanov et al. 2018).

In somatic cells, it is unlikely that cohesin acetylation by ESCO2 after DNA replication is complete would lead to deleterious effects. We have shown previously that both ESCO1 and ESCO2 are able to mediate cohesin acetylation in *Xenopus* egg extract (Song et al. 2012; Lafont, Song, and Rankin 2010). ESCO2 added to extract in which DNA replication is blocked, or after replication is complete, still readily promotes cohesin acetylation (Song et al. 2012). In somatic cells, cohesin is acetylated throughout interphase by ESCO1 (Song et al. 2012). Thus although ESCO2 remains present in G2, we have no evidence that its activity during G2 is deleterious. It is likely that once the MCM complex is unloaded, ESCO2 can no longer promote new sister cohesion, and thus ESCO2 may act more non-specifically at this time. It is also possible that ESCO2 could be re-engaged to modulate cohesin function during post-replication DNA repair.

## Experimental procedures

### Gels and immunoblots

Egg extract samples were diluted in sample buffer (1:10) and loaded onto a 7–15% gradient SDS-PAGE gel to resolve proteins. Gels were cut near the 25 kDa marker and the lower fragment was stained with a colloidal Coomassie stain. Proteins were transferred from the remainder of the gel to nitrocellulose membrane using the Trans-Blot Turbo (Bio-Rad). Membranes were incubated in a 5% milk in 1× Tris-buffered saline, 0.05% Tween 20 (TBST) for 45 min at room temperature, probed using primary antibodies overnight at 4 °C, washed three times in 1× TBST, probed with horseradish peroxidase (HRP)-conjugated secondary antibodies for 45 min at room temperature, washed three times in 1× TBST, and one time with 1× TBS. Signals were detected with chemiluminescent substrate (Licor Biosciences) and imaged with the Azure C600 CCD imager (Azure Biosystems). Intensity measurements were made using Image Studio Lite (Licor Biosciences) using median background subtraction and top/bottom setting.

### Antibodies

P-Chk1 antibodies were diluted in 5% BSA in 1× TBST with 0.1% NaN_3_, and all other primary antibodies were diluted in TBST, 5% milk powder with 0.1% NaN_3_. Goat anti-rabbit and donkey anti-mouse secondary antibodies were used diluted in TBST with 5% milk powder (Table 1).

**Table 1:** Antibodies used in current study

Antigen	Antibody source	Number	Dilution	Reference
*β*-tubulin	Development Studies Hybridoma Bank	E7	1:2000	(Chu and Klymkowsky 1989)
GFP	Millipore-Sigma	SAB2702211	1:5000	
hESCO2	Bethyl Labs	A301-689A	1:3000	
P-H3S10	Upstate	06-570	1:1000	
P-Chk1	Cell Signaling	2348	1:1200	
hSMC3	Lab made	OMRF160	1:1000	(Song et al. 2012)
xESCO2	Lab made	OMRF161	1:2000	(Lafont, Song, and Rankin 2010)
xMCM10	Walter lab		1:5000	(Wohlschlegel et al. 2002)
xCdt1	Walter lab		1:2000	(Arias and Walter 2005)
Donkey α-mouse (HRP conjugated)	Jackson ImmunoResearch	715035151	1:5000	
Goat α-rabbit (HRP conjugated)	Thermo Fisher Scientific	31460	1:10,000	

### Xenopus egg extracts

Egg extracts were prepared according to established protocols (Gillespie, Gambus, and Blow 2012). *Xenopus laevis* eggs were collected in 1× MMR (5 mM HEPES, pH 7.7; 100 mM NaCl; 2 mM KCl; 1 mM MgCl_2_; 2 mM CaCl_2_; 0.1 mM EDTA), dejellied in a 2% cysteine in water containing 1 mM EGTA, and washed 4–5 times with XBE2 (100 mM KCl; 2 mM MgCl_2_; 0.1 mM CaCl_2_; 1.7% sucrose; 5 mM K-EGTA; 10 mM HEPES, pH 7.7). Eggs were then transferred to fill 14 × 89 mm round-bottom tubes (Beckman Coulter, 331372) supplemented with protease inhibitors (10 μg/ml each of leupeptin, pepstatin, and chymostatin, final). The eggs were packed by centrifugation (1000 RPM for 1 min at 4 °C) in a Beckman JS13.1 rotor. Excess buffer was removed and the eggs were crushed by centrifugation at 10,000 RPM for 10 min at 4 °C in the same rotor. The cytosolic layer was removed via side puncture 16G needle, and supplemented with protease inhibitors (10 μg/ml each of leupeptin, pepstatin, and chymostatin), cytochalasin B (10 μg/ml), 15% LFB1/50 (40 mM HEPES, pH 8.0; 20 mM K_2_HPO_4_/KH_2_PO_4_, pH 8.0; 2 mM MgCl_2_; 1 mM EGTA; 2 mM DTT; 10% sucrose; 50 mM KCl), transferred to round-bottom tubes (Beckman Coulter, 344057), and spun at 30,000 RPM in a Beckman Sw55 rotor for 20 min at 4 °C. The lipid plug was pushed aside and the cytosol, including floating membrane layer, was collected using a pipette tip. The extract was supplemented with 2% v:v glycerol and snap-frozen in 100 μl aliquots using liquid nitrogen and stored at −80 °C.

Prior to use, extracts were quickly thawed in hand, set on ice, and supplemented with freshly prepared 35× stock of energy mix (650 mM phosphocreatine, 130 μg/ml creatine phosphokinase, and 65 mM ATP) made from frozen components. To release extract from CSF arrest, a freshly made 10 mM CaCl_2_ solution was added (0.4 mM final in extract) and extracts were incubated in a 20 °C water bath for 30 min. Where indicated, cycloheximide (Sigma Aldrich) was added from a 10 mg/ml stock to 250 μg/ml final, prior to the addition of CaCl_2_. Aphidicolin (VWR) was added to 100 μg/ml final from a 10 mg/ml stock solution following CSF release, before sperm addition. Purified H6-p27 protein at 1 mg/ml was added to egg extracts (20 μg/ml final) following CSF release and before sperm addition, as previously (Song et al. 2012). CSF release was confirmed in all experiments by monitoring nuclear morphology. To do this, 0.5 μl of extract with nuclei was placed in the center of 4 μl of Quick Fix (1XMMR, 1 μg/ml DAPI, 60% glycerol, 11% formaldehyde), a coverslip was added, and an upright epifluorescence microscope equipped with phase contrast optics was used to monitor for the presence of nuclear envelopes. Condensed mitotic chromatids and the absence of nuclear envelope were used to confirm CSF arrest. For chromatin binding assays, 10 μl aliquots of the nuclear assembly reaction were collected, diluted in 5 volumes of ice-cold ELB (10 mM HEPES, pH 7.7, 50 mM KCl, 2.5 mM MgCl_2_, 250 mM sucrose), and spun through a 150 μl cushion (ELB containing 0.5 M sucrose) preloaded in a 0.4 ml capless tubes (Evergreen scientific), resuspended in 60 μl ELB containing 0.6% Triton X-100, spun through a second cushion, and resuspended in sample buffer for SDS-PAGE analysis.

### Sperm nuclei

Preparation of demembranated sperm nuclei was as previously described (Chan and Forbes 2006). Briefly, freshly isolated testes were minced and washed in buffer X (10 mM HEPES pH 7.5, 80 mM KCl, 15 mM NaCl, 5 mM MgCl_2_, 1 mM EDTA, 200 mM sucrose), vortexed, and spun with mild centrifugation (10 s at 1000 RPM), repeating until the supernatant was clear. Supernatants were combined and centrifuged twice (50 s at 1500 RPM and 10 min at 4000 RPM at 4 °C). The pellet was then resuspended in buffer X and layered on a sucrose gradient and centrifuged 25 min at 33,000 RPM at 4 °C. The sperm pellet was again resuspended in buffer X and centrifuged (10 min at 5000 RPM at 4 °C), resuspended in buffer X mix #1 (buffer X, 0.4% Triton X-100, and LPC), and incubated while rotating at 4 °C for 30 min. The resulting solution is layered over a sucrose step gradient and centrifuged (10 min at 2100 RPM at room temperature), resuspended in buffer X mix #2 (buffer X, 3% BSA and LPC), and centrifuged (10 min at 2100 RPM at room temperature) twice, and resuspended in a final buffer X mix (buffer X, 3% BSA, LPC, and 1 mM DTT). Sperm concentration was determined using a hemocytometer, and stored in 10 μl aliquots at −80 °C. To damage sperm nuclei, a 5 μl drop of ice-cold sperm (1.2 × 10^5^/μl) was deposited on parafilm at room temperature and irradiated in a Stratalinker 1800 (Stratagene) with ~70 μJ/m^2^ UV. Control sperm were left on the bench on parafilm at room temperature for 5 min.

### Cell culture and cell line construction

Cells were routinely cultured in Dulbecco’s modified Eagle’s medium (DMEM, Corning), supplemented with 10% fetal bovine serum (FBS, R&D Systems), and maintained at 37 °C in 5% CO_2_. Cell lines with a doxycycline-inducible NLS-GFP-tagged ESCO2 transgene were generated using HeLa Flp-In T-Rex ESCO1 KO cells as previously (Alomer et al. 2017). To do this, an siRNA resistant derivative of an ESCO2 cDNA (the sequence CGAGTGATCTATAAGCCAA was modified to CGTGTCATTTACAAACCTA) was cloned into a pcDNA5/FRT-derived vector (Life Technologies) containing NLS-GFP. This plasmid was co-transfected with a plasmid encoding the FLP recombinase (pOG44, Invitrogen) using Lipofectamine 2000 (Invitrogen) according to the manufacturer’s instructions. Cells containing the integrated transgene were selected using 200 μg/ml hygromycin B (Gold Biotechnology), single colonies were isolated using trypsin-soaked filter paper, and screened for transgene expression by immunoblot and flow cytometry. Transgene expression was induced with 24-h incubation in media supplemented with 2 μg/ml doxycycline (VWR). SiRNA-mediated depletion of endogenous ESCO2 was done with 20 nM siRNA (Dharmacon, J-025788-09, target: CGAGUGAUCUAUAAGCCAA) using Lipofectamine RNAiMAX (Invitrogen) according to the manufacturer’s instructions in Opti-MEM serum free medium (Invitrogen). Following 12 h in transfection mix, the media was replaced with fresh standard medium supplemented with 2 μg/ml of doxycycline, cells were incubated for an additional 24 h, and then processed for immunoblot.

### Flow cytometry

To analyze live HeLa cells by flow cytometry, Hoechst 33342 (4 μg/ml) was added to the media for 45 min prior to harvesting. The media was collected and the cells were washed with PBS + 4 μg/ml Hoechst, harvested with trypsin, and resuspended in reserved media. The cells were centrifuged at 1500 RPM for 5 min, resuspended in PBS + 4 μg/ml Hoechst 33342, and run on a FACSCelesta flow cytometer (BD Biosciences). Samples were analyzed using FlowJo v10.5.3 (TreeStar). Single cells were identified using the forward and side scatter (Supplementary Fig. 4a), and the Cell Cycle univariate modeling tool (FlowJo, Watson Pragmatic algorithm) based on DNA content was used to assign cell cycle phases to each dataset (Supplementary Fig. 4b). S phase was further subdivided into early and late populations by imposing a gate at the midpoint of DNA content within the S phase group (Supplementary Fig. 4c). Prism Software (Graph-Pad Software) was used normalize the fluorescence intensity values to the average G1 intensity for each of the three biological replicates.

### Live-cell imaging

Cells induced to express GFP-ESCO2 overnight were imaged in Opti-MEM (Gibco) supplemented with 2 μg/ml of doxycycline and 250 nM siR-DNA (Cytoskeleton, Inc.) at 37 °C in a 5% CO_2_ atmosphere using a stage top incubator (Tokai Hit). Images were collected every 10 min for 48 h using a 20× S Plan Fluor ELWD objective lens on a Nikon Eclipse TE2000-E equipped with the Perfect Focus and triggered acquisition, with a Hamamatsu Orca-Flash4.0 CMOS camera and Lumencor light engine light source. Images were analyzed using NIS Elements software. Beginning with metaphase figures, the background-subtracted sum cellular GFP intensity of each of 20 cells was measured every 40 min until the subsequent metaphase of the two daughter cells and plotted, each normalized to the signal intensity of the maternal cell. The time (*x*) axis for each cell was normalized to individual cell cycle lengths, so that measurements at M1 and M2 for all cells are aligned for direct comparison.

### Statistical analysis

Prism v9.3 (Graph-Pad Software) was used to plot data and perform statistical analysis. For analyses with multiple comparisons, we used an ordinary one-way ANOVA and Tukey’s multiple comparison test, with a single pooled variance. For analysis of flow cytometry data, the fluorescence intensity values were exported into Prism and normalized to the average G1 intensity for each of the three biological replicates, which were then overlaid in a Superplot (Lord et al. 2020).

## Supplementary information

Supplementary Figure 1. Esco2 remains associated with chromatin after PCNA unloading. The chromatin association of ESCO2 and PCNA were monitored over the course of DNA replication in extract released from CSF arrest. **a**. Samples collected at the indicated times were analyzed by immunoblot. **b.** The experiment shown in a was performed three times, and the signals for ESCO2 and PCNA were normalized to ORC2. Error bars: Standard deviation. (PNG 214 kb)

High resolution image (TIFF 360 kb)

Supplementary Figure 2. MCM10 is unresponsive to DNA damage signaling in extracts. Immunoblot analysis. Reactions used in Fig. 3 with the indicated modifications. UV: sperm were UV treated before they were added to the extract. Aphid: the DNA replication inhibitor aphidicolin was added to the extract before the addition of nuclei. p27: Recombinant p27 protein was added to the extract before the addition of nuclei. Samples were collected at the indicated times and probed by immunoblot for MCM10. Antibody specific for phosphorylated Chk1 kinase (pChk1) was used to confirm DNA damage signaling. Solid outlines denote membrane fragments that were processed separately. Dotted lines denote where blot images were cropped. (PNG 214 kb)

High resolution image (TIFF 405 kb)

Supplementary Figure 3. Relative protein expression during early development. **a.** Illustration of proteins that comprise the CUL4-DDB1-DCAF1^VprBP^ complex. **b.** Plot of relative protein expression for each component shown in B*.* For the genes that have expression from homeologous copies in the allotetraploid *Xenopus laevis*, the shorter and longer homeologs are signified with .S or .L respectively. Colors are coordinated with illustration in A. Dtl is an alternate name for DCAF2, the specificity factor for Cdt1 degradation (Havens and Walter 2009). This graph illustrates changes in the levels of each protein during early development, but is not meant for direct comparison between proteins, which may not be valid. Data were retrieved from https://www.xenbase.org/gene/geneExpressionChart.do?method=drawProtein and are described in (Peshkin et al. 2019). (PNG 403 kb)

High resolution image (TIFF 555 kb)

Supplementary Figure 4. Gating strategy and cell cycle assignment for flow cytometry analysis. **a.** Selection of single-cell population to exclude debris and cell clumps. SSC-A = side scatter area, FSC-A = forward scatter area. **b.** Cell cycle distribution of the single-cell population selected in A following analysis with the FlowJo Cell Cycle tool. **C.** S phase cells identified in B were bisected into early and late S phase at the midpoint of DNA content. (PNG 352 kb)

High resolution image (TIFF 550 kb)

Video S1. Time-lapse movie of HeLa GFP-ESCO2 expressing cell from which stills shown in Fig. 4 were extracted. Differential Interference Contrast (DIC) left and GFP (right). Elapsed time (hours:minutes) is shown at the top left. (AVI 2464 kb)

Video S2. Time-lapse imaging of GFP-ESCO2 expressing cells, large field of view. Differential Interference Contrast (DIC) (left) and GFP (right). White arrow heads indicate examples of metaphase cells that undergo cell division over the course of the movie. Elapsed time (hours:minutes) is shown at top left. (AVI 15720 kb)

## Data Availability

All data are included with the manuscript. Any additional details will gladly be provided upon request.

## Abbreviations

APC: Anaphase promoting complex
Cdh1: Cdc20 homolog 1
Cdt1: Chromatin licensing and DNA replication factor 1
Chk1: Checkpoint kinase 1
CRL4: Cullin-RING ubiquitin ligase complex 4
CSF: Cytostatic factor
CUL4: Cullin 4
DCAF1: DDB1 and CUL4 associated factor 1
DDB1: Damage specific DNA binding protein 1
Dox: Doxycycline
Eco1p: Establishment of cohesion protein 1
ESCO1: Establishment of cohesion 1
ESCO2: Establishment of cohesion 1 homolog 2
FoxM1: Forkhead box M1
MCM: Minichromosome maintenance
NF2: Neurofibromatosis type 2
ORC2: Origin recognition complex subunit 2
PCNA: Proliferating cell nuclear antigen
PIP: PCNA-interacting protein
SAMHD1: SAM and HD domain containing deoxynucleoside triphosphate triphosphohydrolase 1
SMC3: Structural maintenance of chromosomes 3
VprBP: Viral protein R binding protein
WAPL: Wings apart-like protein homolog
