# Supplementary figures and images for: The cohesin modifier ESCO2 is stable during DNA replication
Source: Chromosome Res. 2023 Jan 28;31(1):6. doi: 10.1007/s10577-023-09711-1 (PMC9884251; doi:10.1007/s10577-023-09711-1)

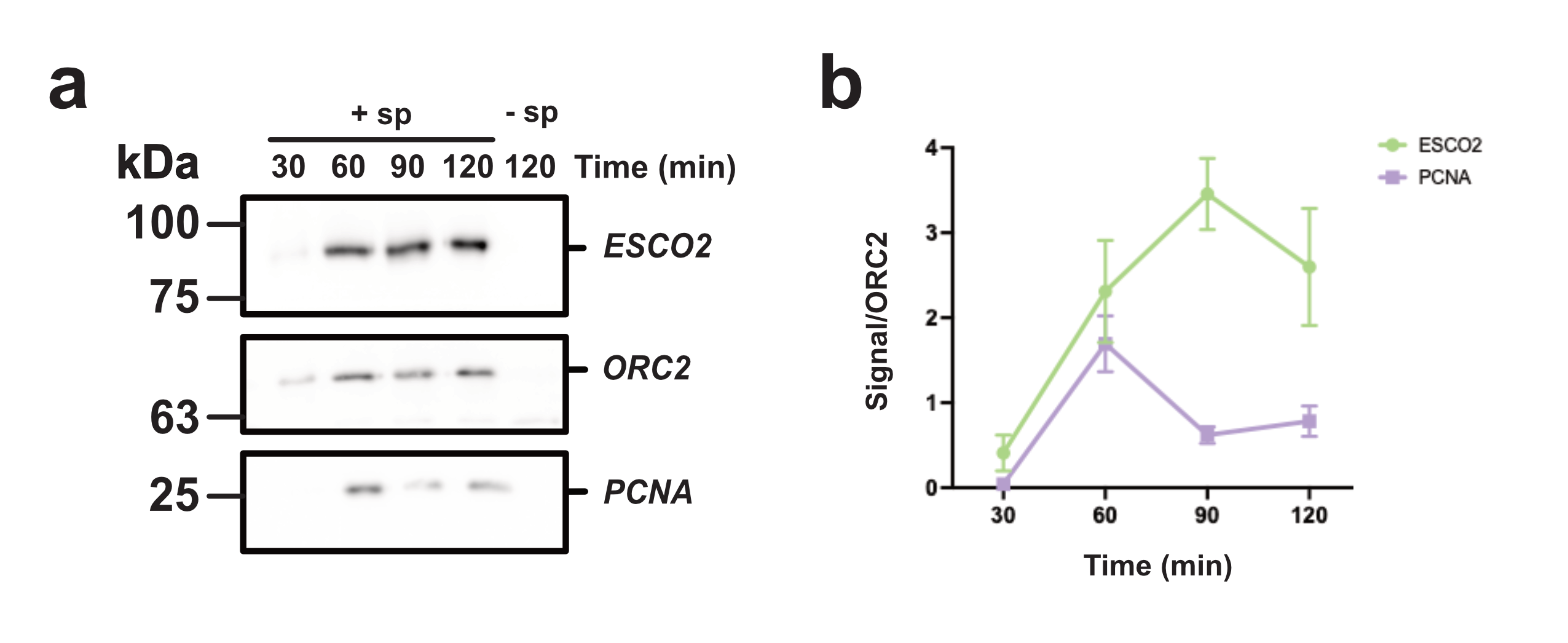

Supplement: Supplementary file 1 — Esco2 remains associated with chromatin after PCNA unloading. The chromatin association of ESCO2 and PCNA were monitored over the course of DNA replication in extract released from CSF arrest. a. Samples collected at the indicated times were analyzed by immunoblot. b. The experiment shown in a was performed three times, and the signals for ESCO2 and PCNA were normalized to ORC2. Error bars: Standard deviation. (PNG 214 kb) [file 10577_2023_9711_Fig5_ESM.png]

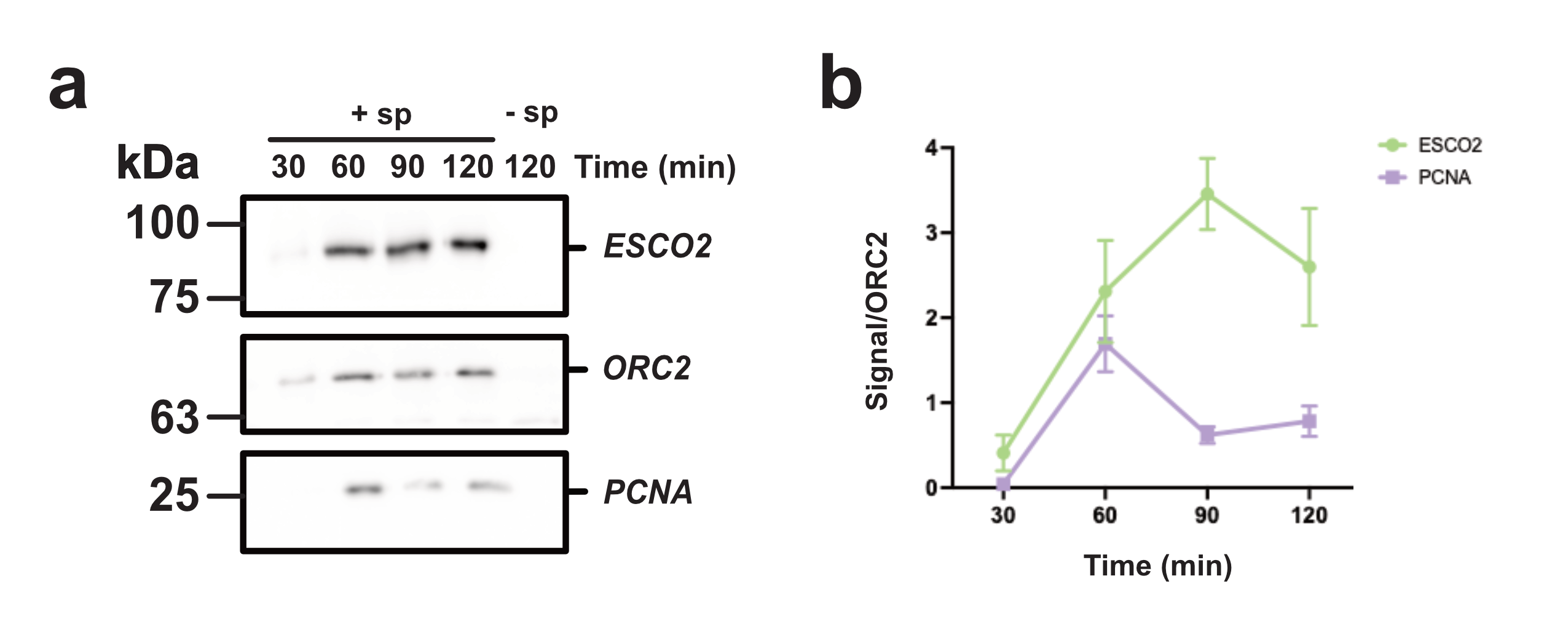

Supplement: Supplementary file 2 — High resolution image (TIFF 360 kb) [file 10577_2023_9711_MOESM1_ESM.tiff]

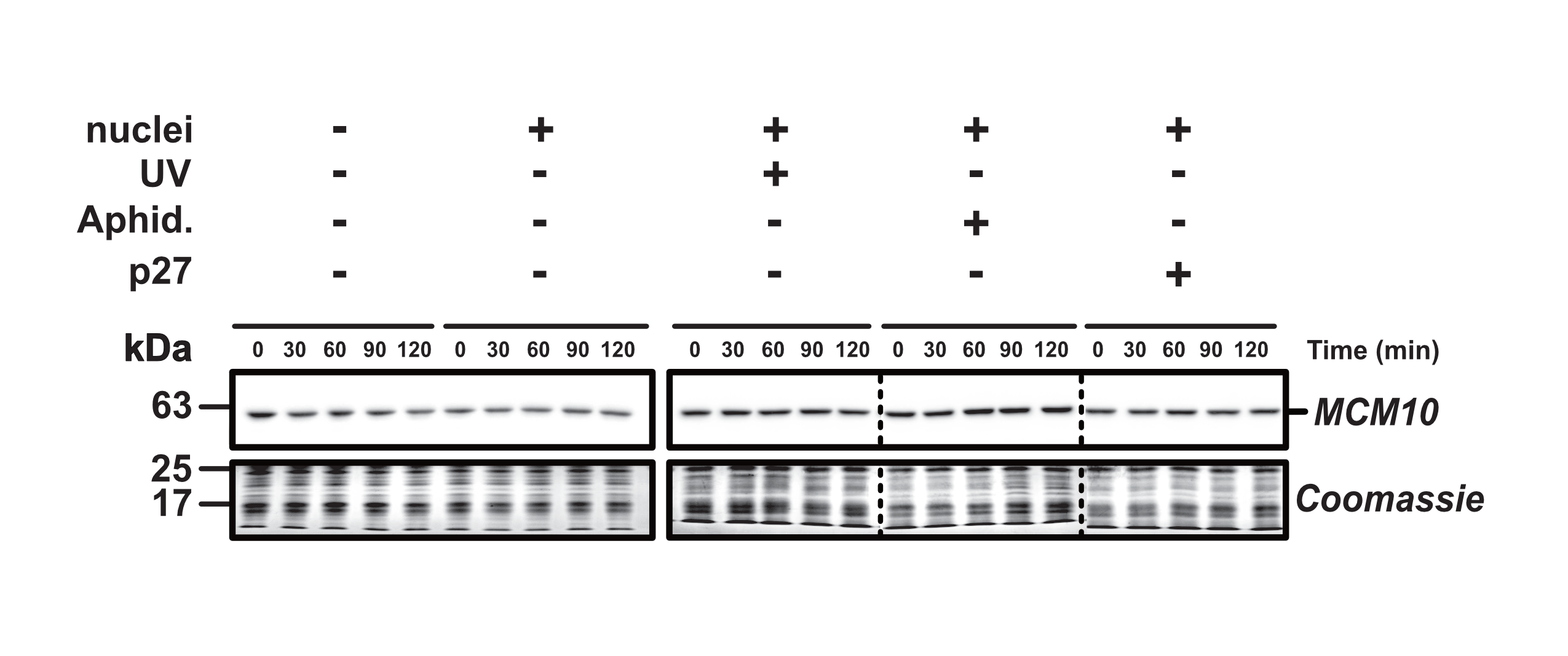

Supplement: Supplementary file 3 — MCM10 is unresponsive to DNA damage signaling in extracts. Immunoblot analysis. Reactions used in Fig. 3 with the indicated modifications. UV: sperm were UV treated before they were added to the extract. Aphid: the DNA replication inhibitor aphidicolin was added to the extract before the addition of nuclei. p27: Recombinant p27 protein was added to the extract before the addition of nuclei. Samples were collected at the indicated times and probed by immunoblot for MCM10. Antibody specific for phosphorylated Chk1 kinase (pChk1) was used to confirm DNA damage signaling. Solid outlines denote membrane fragments that were processed separately. Dotted lines denote where blot images were cropped. (PNG 214 kb) [file 10577_2023_9711_Fig6_ESM.png]

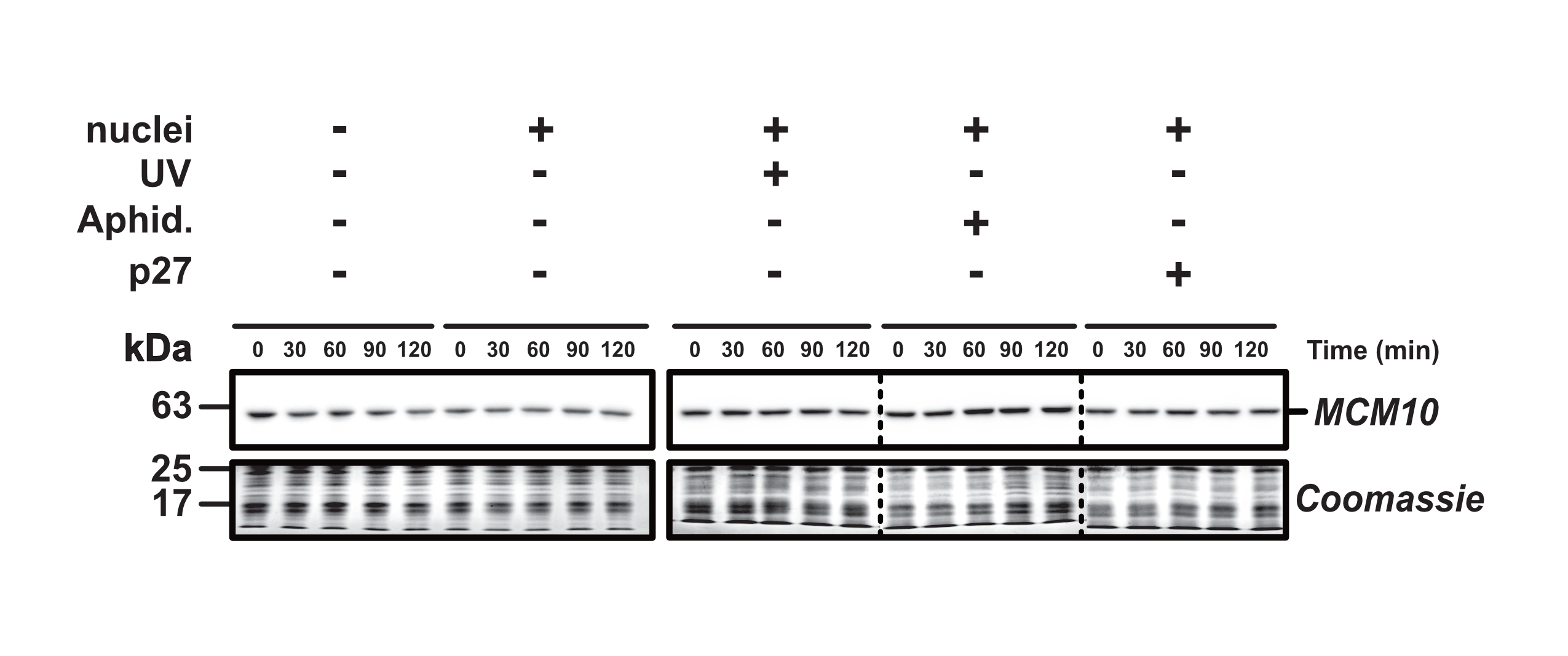

Supplement: Supplementary file 4 — High resolution image (TIFF 405 kb) [file 10577_2023_9711_MOESM2_ESM.tiff]

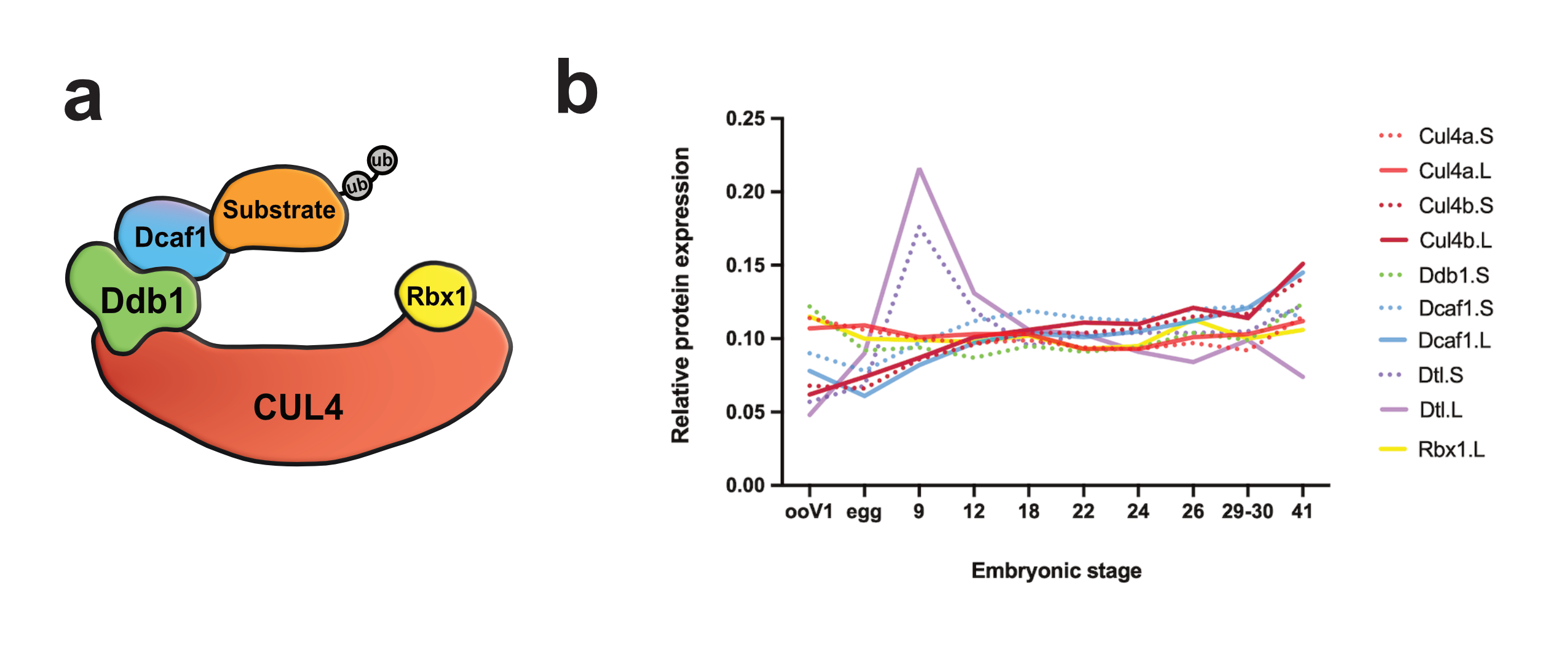

Supplement: Supplementary file 5 — Relative protein expression during early development. a. Illustration of proteins that comprise the CUL4-DDB1-DCAF1VprBP complex. b. Plot of relative protein expression for each component shown in B. For the genes that have expression from homeologous copies in the allotetraploid Xenopus laevis, the shorter and longer homeologs are signified with .S or .L respectively. Colors are coordinated with illustration in A. Dtl is an alternate name for DCAF2, the specificity factor for Cdt1 degradation (Havens and Walter 2009). This graph illustrates changes in the levels of each protein during early development, but is not meant for direct comparison between proteins, which may not be valid. Data were retrieved from https://www.xenbase.org/gene/geneExpressionChart.do?method=drawProtein and are described in (Peshkin et al. 2019). (PNG 403 kb) [file 10577_2023_9711_Fig7_ESM.png]

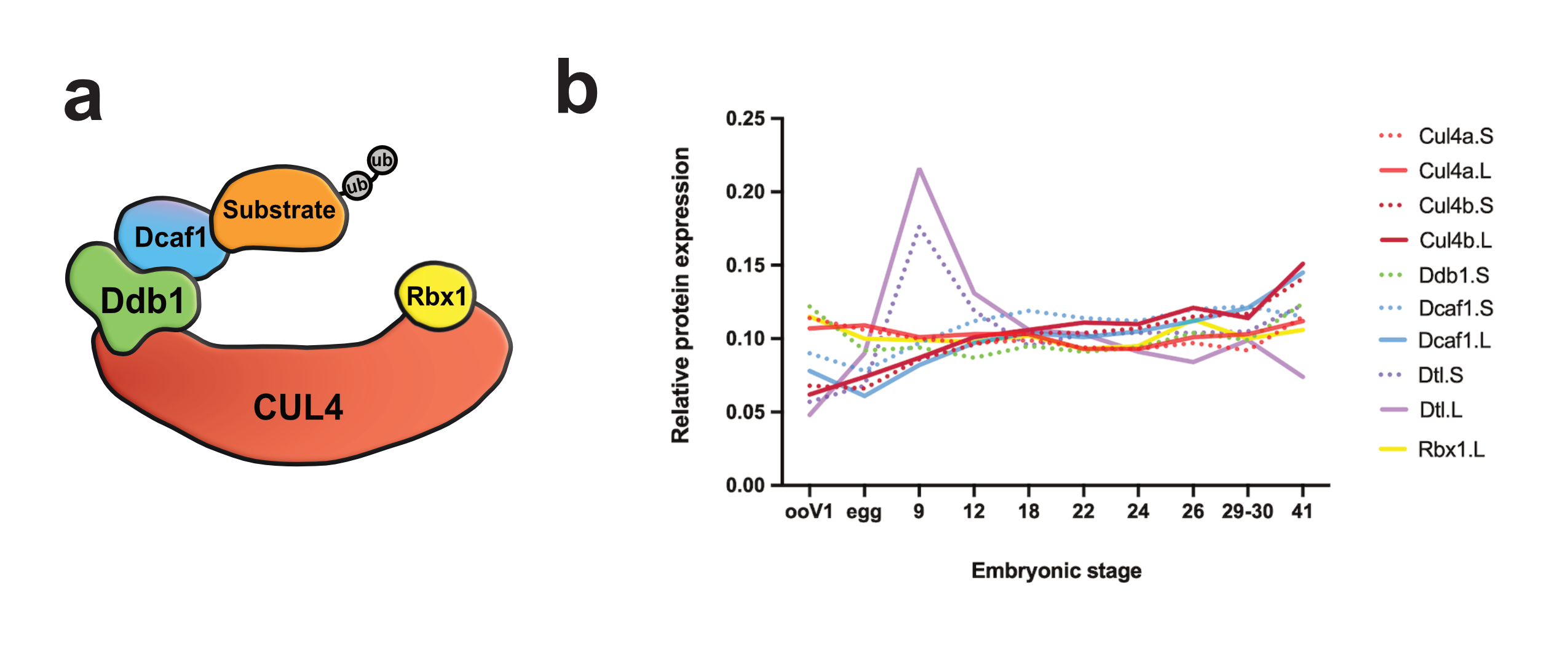

Supplement: Supplementary file 6 — High resolution image (TIFF 555 kb) [file 10577_2023_9711_MOESM3_ESM.tiff]

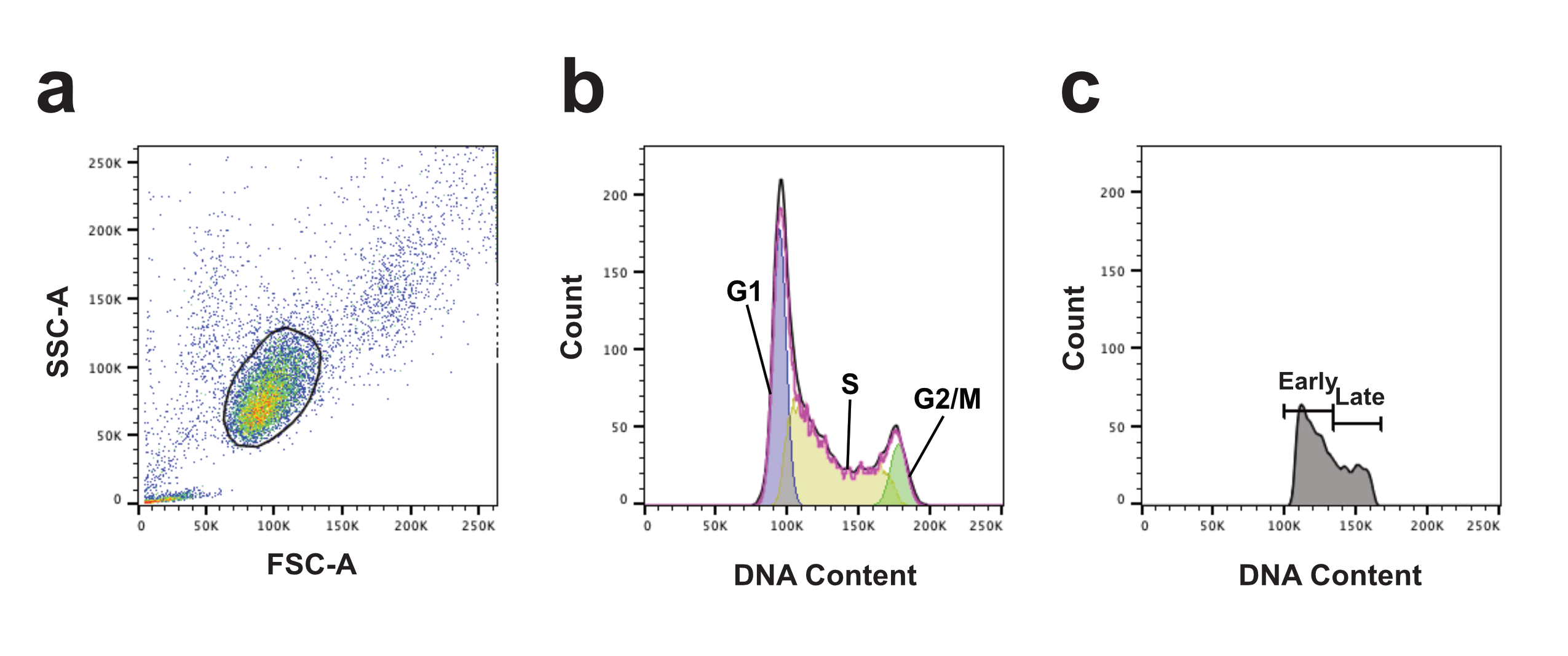

Supplement: Supplementary file 7 — Gating strategy and cell cycle assignment for flow cytometry analysis. a. Selection of single-cell population to exclude debris and cell clumps. SSC-A = side scatter area, FSC-A = forward scatter area. b. Cell cycle distribution of the single-cell population selected in A following analysis with the FlowJo Cell Cycle tool. C. S phase cells identified in B were bisected into early and late S phase at the midpoint of DNA content. (PNG 352 kb) [file 10577_2023_9711_Fig8_ESM.png]

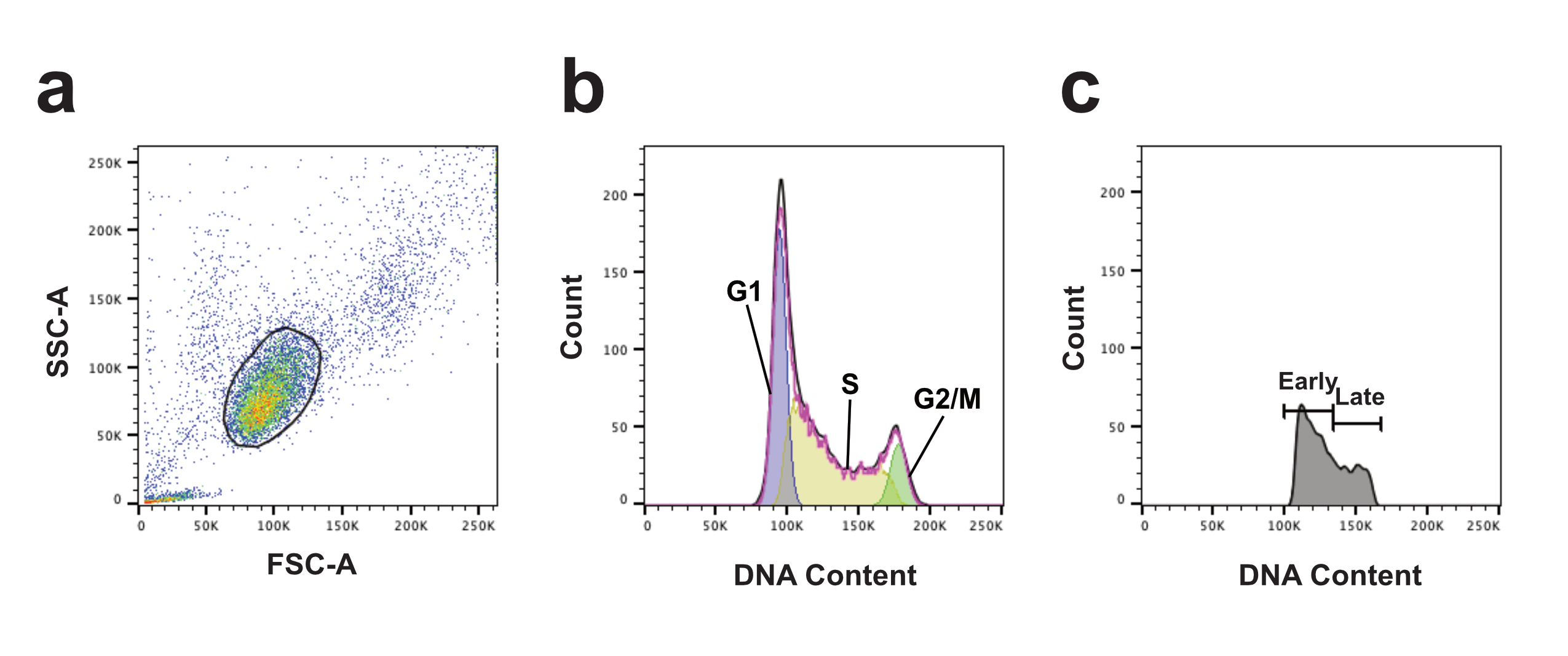

Supplement: Supplementary file 8 — High resolution image (TIFF 550 kb) [file 10577_2023_9711_MOESM4_ESM.tiff]
